# A pan-cancer analysis of the role of HOXD1, HOXD3, and HOXD4 and validation in renal cell carcinoma

**DOI:** 10.18632/aging.205116

**Published:** 2023-10-12

**Authors:** Lumin Wang, Xiaofei Wang, Haifeng Sun, Wenjing Wang, Li Cao

**Affiliations:** 1 Department of Gastroenterology, The Second Affiliated Hospital of Xi’an Jiaotong University, Xi’an, Shaanxi 710004, P.R. China; 2 Key Laboratory of Environment and Genes Related to Diseases, Xi’an Jiaotong University Health Science Center, Xi’an, Shaanxi 710061, P.R. China; 3 The Third Department of Medical Oncology, Shaanxi Provincial Cancer Hospital Affiliated to Medical College of Xi’an Jiaotong University, Xi’an, Shaanxi 710065, P.R. China; 4 Department of Hepatobiliary Surgery, The First Affiliated Hospital of Xi’an Jiaotong University, Xi’an, Shaanxi 710061, P.R. China

**Keywords:** HOXDs, pan-cancer, prognostic biomarker, immune infiltration, KIRC

## Abstract

HOXD1, HOXD3, and HOXD4 are members of the HOXD genes family and are related to tumorigenesis of the tumor. However, whether HOXDs (1, 3, 4) have a crucial role across pan-cancer is still unknown. HOXD1, HOXD3, and HOXD4 expressions were analyzed using public databases in 33 types of tumors. The UCSC Xena website was carried out to investigate the relationship between the expression of genes and the progress of cancers. The biological functions of HOXD3 were tested by colony forming, transwell, wound healing, and xenograft assay *in vitro* and *in vivo*. GSEA was used to identify the associated cancer hallmarks with HOXDs expression. Immune cell infiltration analysis was applied to verify the immune cell infiltrations related to genes. The results showed HOXD1, HOXD3, and HOXD4 co-low expressed in BRCA, COAD, KICH, KIRC, KIRP, READ, and TGCT. In the KIRC, all of HOXDs expression was connected with tumor stage and histological grade. Upregulation of HOXDs was associated with improved OS, DSS, and PFI. Down-expression of HOXD3 induced cell proliferation, migration, and invasion *in vivo* and *in vitro*. In addition, HOXDs were connected with immune-activated hallmarks and cancer immune cell infiltrations. These findings demonstrated that HOXDs may be indicative biomarkers for the prognosis and immunotherapy in pan-cancer.

## INTRODUCTION

Renal cell carcinoma (RCC) is one of the most frequent causes of cancer-related mortality worldwide [1]. Among all histological subtypes of RCC, kidney renal clear cell carcinoma (KIRC) accounts for about 75% of all RCC. Although the diagnostic technology and surgical treatment of KIRC have gradually improved, patient prognosis remains poor [2]. One important reason is the lack of targetable molecules for diagnosis and treatment. Therefore, studying the molecular mechanism underlying KIRC is of great importance for the development of early diagnosis and novel treatment strategies.

HOX genes are highly conserved homeotic genes, which contain 39 members and include four gene clusters: HOXA, HOXB, HOXC, and HOXD [3, 4]. Previous research showed that the dysregulation of HOX genes is identified in various cancers, including GC [5], breast cancer [6], and so on, where they serve as oncogenes or as tumor suppressors to exert their function [7]. The HOXD family comprises nine genes, namely, HOXD1, HOXD3, HOXD4, HOXD8, HOXD9, HOXD10, HOXD11, HOXD12, and HOXD13. A growing number of studies show that HOXD family genes participated in modulating the progression of various biological processes, such as apoptosis, metastasis, angiogenesis, and differentiation. Hui Liu reported that overexpression of HOXD4 could lead to poor clinical outcomes in gastric adenocarcinoma [8]. In breast cancer, high expression of HOXD8 suppressed the proliferation, metastasis, and invasion of breast cancer by inhibiting the ILP2 expression [9]. HOXD10 expression was significantly associated with clinical and pathological indicators in patients with colorectal cancer [10], endometrial cancer [11], hepatocellular carcinoma [12], bladder cancer [13]. Above all, emerging evidence suggests that HOXDs play an essential role in diverse biological processes and its dysregulation is associated with tumorigenesis, either as an oncogene or tumor suppressor.

Based on sequence homology and HOXD family position in the cluster, the HOXD1, HOXD3, and HOXD4 expressions are connected with the proper patterning of the head and neck vertebrae [14]. In the research of tumor progression and migration, the expression of HOXD1 is involved in cell proliferation, cell cycle, and the TGF-β signaling in KIRC [15]. Upregulation of HOXD4 was markedly correlated with poorer prognosis of gastric adenocarcinoma patients [8]. Moreover, in our previous research, we found that HOXD3 could target the promoter region of ITGA2 [16] to induce liver cancer cell progression, invasion, metastasis, and angiogenesis. Whereas, the biological and molecular mechanisms of HOXD1, HOXD3, and HOXD4 in KIRC remain unclear.

The current study conducted the bioinformatics database to analyze the expression of HOXD1, HOXD3, and HOXD4 in common cancers. Low-expressed HOXD1, HOXD3, and HOXD4 are associated with the poor clinical stage and G stage, and shorter OS of KIRC patients. Tumor Immune Estimation Resource, Single-Cell Analysis, and gene set enrichment analysis (GSEA) were performed to investigate the special functions and mechanisms of the HOXD1, HOXD3, and HOXD4 in cancers.

What’s more, elevated HOXD3 induces the G1 accumulation of the cell cycle, resulting in inhibiting the progression of KIRC. Finally, HOXD3 decreased the invasion and migration of KIRC. Our results suggested that HOXD1, HOXD3, and HOXD4 as tumor suppressor genes, are crucially involved in KIRC progression and metastasis, which may act as potential diagnostic biomarkers for KIRC.

## MATERIALS AND METHODS

### HOXD1, HOXD3, and HOXD4 expression in pan-cancer

The data of the HOXD1, HOXD3, and HOXD4 expression in 33 cancerous tissues and normal tissues were obtained from TCGA (The Cancer Genome Atlas) and GTEx (The Genotype-Tissue Expression) databases, which were downloaded from the University of California Santa Cruz (UCSC) Xena website (https://xena.ucsc.edu). Student’s *t*-test was applied for comparing the differential HOXD1, HOXD3, and HOXD4 expression between tumor and normal tissues.

### Correlation analysis between the HOXD1, HOXD3, and HOXD4 expression and the clinical stage and pathological grades in cancers

The data on clinical stage and grade of cancers were obtained from the UCSC Xena website (https://xena.ucsc.edu). The tumor tissues were mated to different clinical stages and pathological grades, which were used to evaluate the correlated HOXD1, HOXD3, and HOXD4 expression with the clinical progress of various cancers.

### Prognostic value of HOXD1, HOXD3 and HOXD4 expression in pan-cancer

The effect of HOXD1, HOXD3, and HOXD4 expression on prognosis data including overall survival (OS), disease-specific survival (DSS), disease-free interval (DFI), and progression-free interval (PFI) in 33 types of cancers was calculated using the data from TCGA. The Cox regression and Kaplan-Meier (KM) plots were conducted to explore the influence of HOXD1, HOXD3, and HOXD4 on each prognosis type in pan-cancer.

### Relationship between expression of HOXD1, HOXD3, and HOXD4 and immunity

The Tumor Immune Estimation Resource (TIMER) database (http://timer.cistrome.org/) was applied for evaluating the relationship between the expression of HOXD1, HOXD3, and HOXD4 and markers of different subsets of immune cells.

### Single-cell analysis of HOXD1, HOXD3, and HOXD4

Tumor Immune Single-cell Hub (TISCH) web tool was used to reveal the correlations between HOXD1, HOXD3, and HOXD4 expression and infiltrating immune cells. The analysis parameters contain HOXD genes, minor lineage, and various cancers. The HOXD1, HOXD3, and HOXD4 expressions in each cell type were quantified and visualized by a heatmap.

### Gene set enrichment analysis

GSEA was used to explore the relationship between the KEGG pathways and tumor hallmarks associated with HOXD1, HOXD3, and HOXD4. Hallmark gene sets (“h.all.v7.5.1.entrez.gmt”) were obtained from the Broad Institute database.

### Cell lines and cell culture

Three cell lines, 786-O, CAKI-1, and a human tubular epithelial cell line, HK-2 were involved in the study. They were obtained from the Cell Repository of the Shanghai Institute of Life Sciences, Chinese Academy of Sciences. 786-O and HK-2 cells were maintained in RPMI-1640 medium (Thermo Fisher Scientific, Inc., USA) supplemented with 12% fetal bovine serum (Biological Industries, Kibbutz Beit-Haemek, Israel). CAKI-1 was cultured in McCoy’s 5a Modified medium (Thermo Fisher Scientific, Inc.) supplemented with 12% FBS. All cell lines were cultured in a humidified atmosphere containing 5% CO2 at 37°C.

### Quantitative real-time PCR

Total RNA extraction of the cells was isolated using TRIzoL (Invitrogen, USA), and reverse transcription was performed using a cDNA Synthesis Kit (Yeasen, Shanghai, China). qRT-PCR was performed with the SYBR Green PCR kit (Yeasen, Shanghai, China). Quantitation of relative gene expression was calculated using the 2^−ΔΔCt^.

### Western blotting

The transfected cells were lysed with a RIPA buffer. The protein concentration was detected by a BCA protein assay kit (Thermo Fisher Scientific). Total proteins were separated on 10–15% SDS–PAGE gels and removed to PVDF membranes (Amersham, USA). The membranes were incubated with anti-HOXD3 (Santa Cruz Biotechnology, USA, sc-130378), MMP2 (Proteintech, USA, 10373-2-AP), E-Cadherin (Proteintech, 20874-1-AP), N-Cadherin (Cell Signaling Technology, USA, #14215), CDK4 (Proteintech, 11026-1-AP), CyclinD1 (Proteintech, 60186-1-Ig) antibodies overnight at 4°C. After washing, the membranes were incubated with HRP-conjugated secondary antibodies. Signals were detected using enhanced chemiluminescence (FD™FD bio-Dura Ecl Hangzhou, China).

### MTT assay

The treated cells were plated into a 96-well plate. After 24, 48, and 72 h, the cell proliferation was evaluated with a sterile MTT solution (Beyotime, Shanghai, China). The 492 nm wavelength on the enzyme-linked instrument (Molecular Devices, USA) was used to detect the absorbance value.

### Colony formation assay

The transfected cells were plated into 6-well plates at a seeding density of 1000 cells/well and cultured for 14 days. The colonies were fixed with 4% paraformaldehyde and stained with crystal violet. The colonies were imaged and counted.

### Wound-healing assay

Transfected cells were seeded in a six-well plate and subsequently scratched by a 10 μL pipette tip. The wound closure was observed under a microscope (Nikon Corporation, Tokyo, Japan) at 0 h and 36 h. The relative percentage of the wound closure was calculated.

### Cell invasion assay

Transfected ccRCC cells were suspended in a serum-free culture medium and placed in the upper chamber which was coated with Matrigel (BD Biosciences, USA). Then the 600 μl complete medium was added to the lower chambers. Cells migrating into the lower chambers were fixed in 4% paraformaldehyde and stained with 0.5% crystal violet. Finally, the cells were counted under a microscope.

### Xenograft assay

Animal experiments were approved by the Animal Experimentation Ethics Committee of Xi’an Jiaotong University. 4-week-old male nude mice were raised in a pathogen-free SPF room. The treatment CAKI-1 cells (LV-OV-HOXD3-Ctrl and LV-OV-HOXD3) were injected into the bilateral groin of mice. Tumor sizes were examined every 3 d for 28 d. The volume of the tumor was assessed as V = (L × W^2^)/2. Subsequently, the mice were anesthetized with isoflurane/oxygen, and the IVIS Spectrum (Xenogen, USA) was used for *in vivo* bioluminescence imaging. The tumor tissues were frozen for qRT-PCR and western blotting assays.

### Statistical analysis

All experiments were performed at least 3 times. The Student’s *t*-test or one-way ANOVA was used to detect the differences among groups. The statistical analysis was performed in GraphPad Prism 7 software (GraphPad Software, Inc., USA). *P*-values < 0.05 were considered significantly different.

## RESULTS

### HOXD1, HOXD3, and HOXD4 expression in pan-cancer

**Figure 1:**
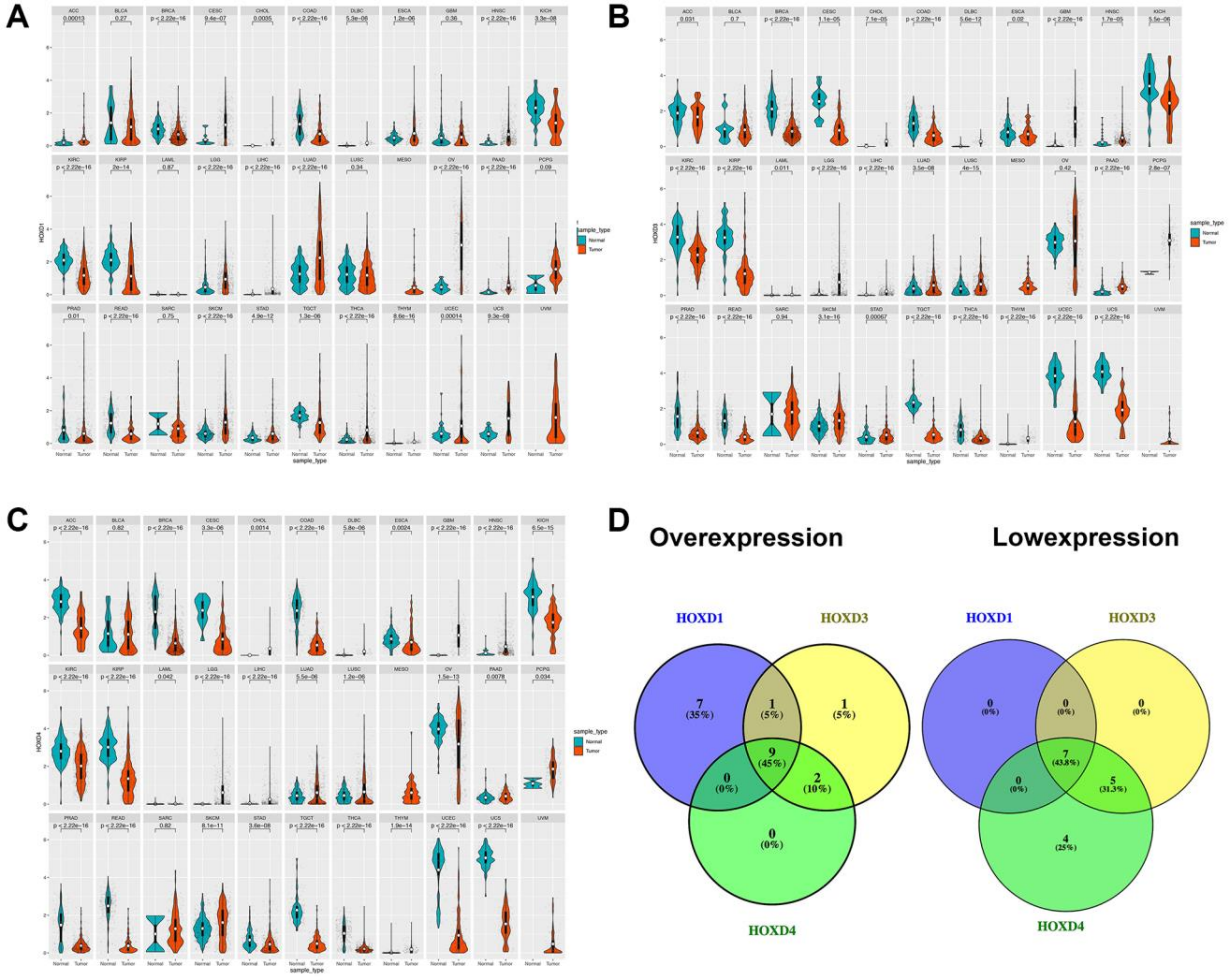
**Differential expression of HOXD1, HOXD3, and HOXD4.** (**A**–**C**) The expression of HOXD1, HOXD3, and HOXD4 in 33 types of cancer respectively. (**D**) HOXD1, HOXD3, and HOXD4 were overexpressed in 9 cancers, which were CHOL, DLBC, HNSC, LGG, LIHC, LUAD, PAAD, SKCM, THYM, and low expressed in BRCA, COAD, KICH, KIRC, KIRP, READ, TGCT.

Analysis of HOXD1, HOXD3, and HOXD4 expression in pan-cancer from the TCGA, and GTEX databases showed that the expression of HOXD1 was markedly enhanced in ACC (Adenoid cystic carcinoma), CESC (Cervical squamous cell carcinoma and endocervical adenocarcinoma), DLBC (Lymphoid neoplasm diffuse Large B-cell Lymphoma), ESCA (Esophageal carcinoma), HNSC (Head and neck squamous cell carcinoma), CHOL (cholangiocarcinoma), LGG (Brain lower grade glioma), LIHC (liver hepatocellular carcinoma), LUAD (Lung adenocarcinoma), OV (Ovarian serous cystadenocarcinoma), PAAD (Pancreatic adenocarcinoma), SKCM (Skin cutaneous melanoma), STAD (Stomach adenocarcinoma), THCA (Testicular germ cell tumors), THYM (Thymoma), UCEC (Uterine corpus endometrial carcinoma), UCS (Uterine carcinosarcoma) (*p* < 0.01). However, the HOXD1 expression was decreased in BRCA (Breast invasive carcinoma), COAD (colon adenocarcinoma), KICH (Kidney chromophobe), KIRC, KIRP (Kidney renal papillary cell carcinoma), READ (Rectum adenocarcinoma), and TGCT (Testicular germ cell tumors) (*p* < 0.01) (Figure 1A).

In the 33 types of cancers, the HOXD3 was increased in CHOL, DLBC, GBM (Glioblastoma multiforme), HNSC, LGG, LIHC, LUAD, LUSC (Lung squamous cell carcinoma), PAAD, PCPG (Pheochromocytoma and paraganglioma), SKCM, STAD, and THYM (*p* < 0.01). Meanwhile, the low-expressed HOXD3 was identified in BRCA, CESC, COAD, KICH, KIRC, KIRP, PRAD, READ, TGCT, THCA, UCEC, UCS (*p* < 0.01) (Figure 1B).

Meanwhile, HOXD4 was significantly overexpressed in CHOL, DLBC, GBM, HNSC, LGG, LIHC, LUAD, LUSC, PAAD, SKCM, and THYM (*p* < 0.01). Inhibition of HOXD4 expression was shown in ACC, BRCA, CESC, COAD, ESCA, KICH, KIRC, KIRP, OV, PRAD, READ, STAD, TGCT, THCA, UCEC, and UCS (*p* < 0.01) (Figure 1C). Based on the above results, we found that HOXD1, HOXD3, and HOXD4 were co-high expressed in 9 cancers. They were CHOL, DLBC, HNSC, LGG, LIHC, LUAD, PAAD, SKCM, and THYM, respectively (Figure 1D). Meantime, HOXD1, HOXD3, and HOXD4 co-inhibition was demonstrated in 7 cancers, such as BRCA, COAD, KICH, KIRC, KIRP, READ, TGCT (Figure 1D).

### HOXD1, HOXD3, and HOXD4 overexpression correlated with the clinical stage of pan-cancer

**Figure 2:**
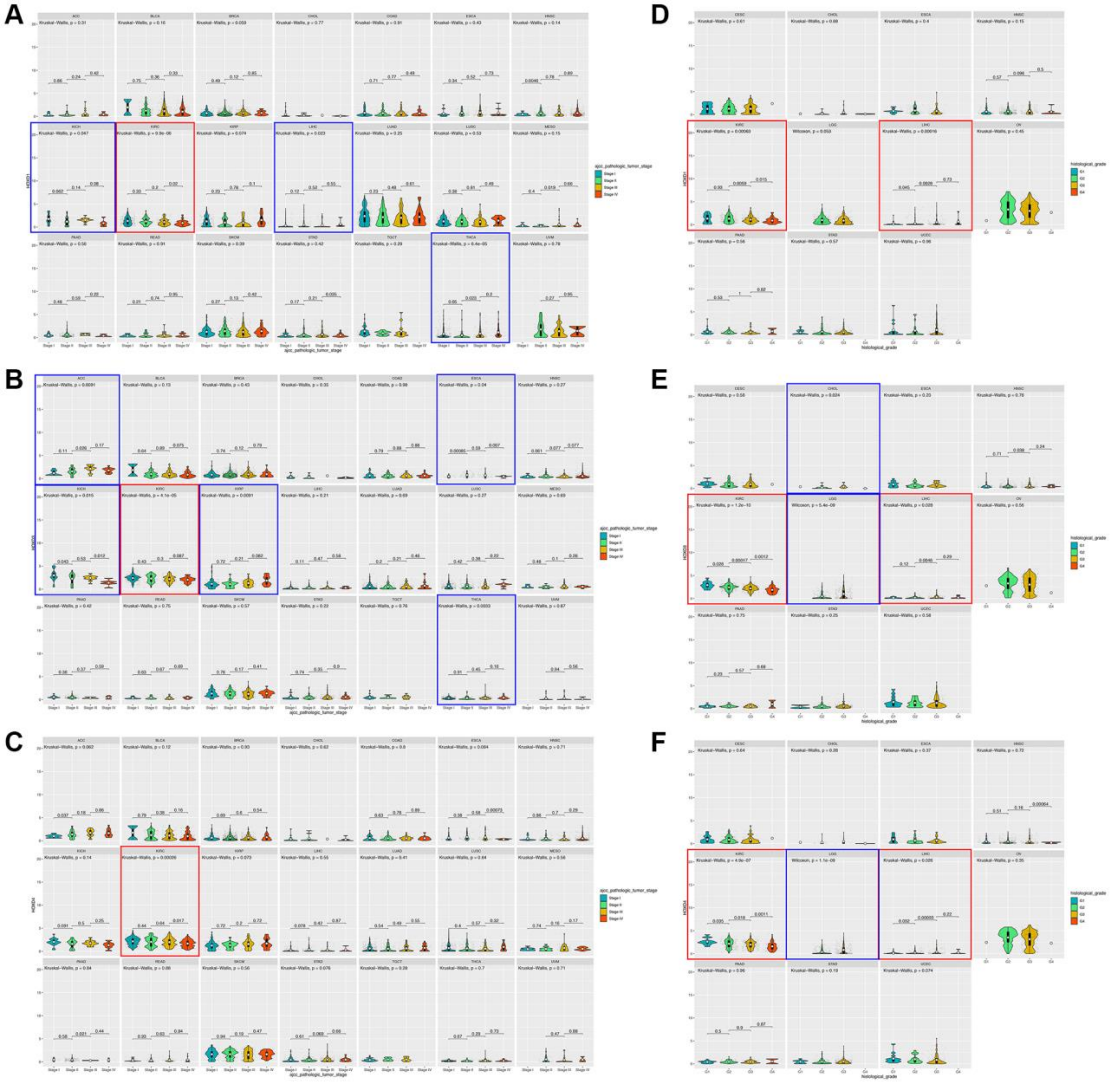
**Association between HOXD1, HOXD3, and HOXD4 expression and tumor stage and histological grade in cancers.** (**A**–**C**) Association between HOXD1, HOXD3, and HOXD4 expression and tumor stage in 21 cancers. (**D**–**F**) Association between HOXD1, HOXD3, and HOXD4 expression and histological grade in 11 cancers.

To assess whether the HOXD1, HOXD3, and HOXD4 overexpression in tumor tissues was related to cancer progression, the result showed that HOXD1 was markedly associated with different pathological stages of cancers, including KICH, KIRC, LIHC, THCA (*p* < 0.05) (Figure 2A). HOXD3 was remarkably associated with the clinical stage of cancers, including ACC, ESCA, KICH, KIRC, KIRP, and THCA (*p* < 0.05) (Figure 2B). Meanwhile, HOXD4 was connected with the clinical stage of cancers, including KIRC (*p* < 0.05) (Figure 2C). Therefore, In the KIRC, all of HOXD1, HOXD3, and HOXD4 expression was significantly correlated with tumor stage (shown in red square frame). Furthermore, the expression of HOXD1, HOXD3, and HOXD4 remarkably correlated with histological grade was demonstrated in 11 cancer types. HOXD1 associated with G stage in KIRC and LIHC (*p* < 0.05) (Figure 2D); HOXD3 in CHOL, KIRC, LGG, and LIHC (*p* < 0.05) (Figure 2E); HOXD4 in KIRC, LGG, and LIHC (*p* < 0.05) (Figure 2F). Therefore, all of HOXD1, HOXD3, and HOXD4 were markedly associated with histological grade in KIRC and LIHC (shown in red square frame).

### HOXD1, HOXD3, and HOXD4 expression connected with prognosis in pan-cancer

**Figure 3:**
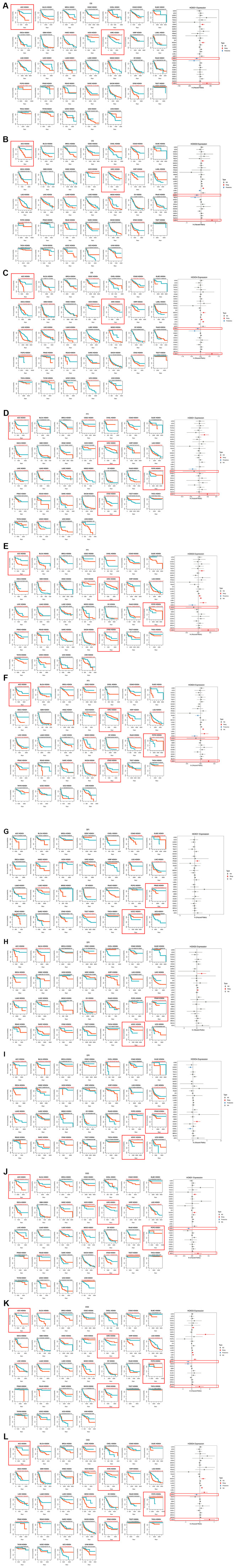
**Association between the HOXD1, HOXD3, and HOXD4 expression and the OS, PFI, DFI, and DSS of cancer patients.** (**A**–**C**) Kaplan-Meier curves showing OS in pan-cancer. Forest plot showing OS after Cox analysis in pan-cancer (right). (**D**–**F**) Kaplan-Meier curves showing PFI in pan-cancer. Forest plot showing PFI after Cox analysis in pan-cancer (right). (**G**–**I**) Kaplan-Meier curves showing DFI in pan-cancer. Forest plot showing DFI after Cox analysis in pan-cancer (right). (**J**–**L**) Kaplan-Meier curves showing DSS in pan-cancer. Forest plot showing DSS after Cox analysis in pan-cancer (right).

The correlation between HOXD1, HOXD3, and HOXD4 and the OS of patients in pan-cancer was analyzed and found that highly expressed HOXD1 was correlated with short OS in ACC, HNSC, MESO, SARC, and STAD (*p* < 0.01) (Figure 3A); HOXD3 in ACC, KIRP, LGG, READ, STAD (Figure 3B) (*p* < 0.01); HOXD4 in ACC, GBM, KIRP, LGG, LUAD, MESO, READ and PRAD (Figure 3C) (*p* < 0.01). Furthermore, overexpression of HOXD1 was connected with long OS in KICH, KIRC, and PCPG (*p* < 0.01); HOXD3 in KICH and KIRC (*p* < 0.01); HOXD4 in KIRC. Above all, increased expression of HOXD1, HOXD3, and HOXD4 is linked with poor OS in ACC, and with long OS in KIRC (shown in red square frame). The results were also observed using Cox regression analysis (Figure 3A–3C).

Furthermore, the expression of HOXD1 was associated with decreased PFI patients in ACC, LIHC, STAD, and UCEC (*p* < 0.01) (Figure 3D); HOXD3 in ACC, KIRP, LGG, PRAD, and STAD (*p* < 0.01) (Figure 3E); HOXD4 in ACC, CESC, DLBC, KIRP, LGG, PRAD, and STAD (*p* < 0.01) (Figure 3F). Meanwhile, HOXD1 was associated with improved PFI in CHOL, KIRC, and PCPG (*p* < 0.01); HOXD3 in KIRC and PCPG (*p* < 0.01); HOXD4 in KIRC and PCPG (*p* < 0.01). So, in the ACC and STAD (shown in red square frame), the high expression of HOXD1, HOXD3, and HOXD4 could lead to poor PFI. Whereas upregulation of HOXD1, HOXD3, and HOXD4 contributed to improved PFI in KIRC and PCPG (shown in red square frame). Consistently, Cox regression results showed that elevated expression of HOXD1, HOXD3, and HOXD4 could lead to poor PFI in ACC, and improved PFI in KIRC (Figure 3D–3F).

Meanwhile, the association of HOXD1 expression with DFI was analyzed, and the results showed that high expression of HOXD1 induced poor DFI in KICH and PRAD (*p* < 0.01) (Figure 3G); HOXD3 in ACC, BLCA (bladder urothelial carcinoma), and PRAD (*p* < 0.01) (Figure 3H); HOXD4 in BLCA, ESCA, KICH, PCPG, and PRAD (*p* < 0.01) (Figure 3I). However, upregulation of HOXD1 was positively associated with DFI in UCEC (*p* = 0.01); HOXD3 in SARC and UCEC (*p* < 0.01); HOXD4 in UCEC (*p* < 0.01). Above all, upregulation of HOXD1, HOXD3, and HOXD4 was correlated with poor DFI in PRAD, and with improved DFI in UCEC (shown in red square frame).

In the research on the connection of HOXD1 with DSS, the results showed that high expression of HOXD1 could lead to short DSS in ACC, MESO, STAD (*p* < 0.01) (Figure 3J); HOXD3 in ACC, BLCA, KIRP, LGG, LIHC, READ, and STAD (*p* < 0.01) (Figure 3K); HOXD4 in ACC, GBM, KIRP, LGG, LUAD, MESO, READ, STAD, and THCA (*p* < 0.01) (Figure 3L). Whereas, upregulation of HOXD1 contributed to improved DSS in KIRC, PCPG, and TGCT (*p* < 0.01); HOXD3 in KICH, KIRC, and PCPG (*p* < 0.01); HOXD4 in KIRC and PCPG (*p* < 0.01). Therefore, upregulation of HOXD1, HOXD3, and HOXD4 was correlated with poor DSS in ACC and STAD, and improved DSS in KIRC and PCPG (shown in red square frame). Consistent with the above results, cox regression results demonstrated that elevated expression of HOXD1, HOXD3, and HOXD4 could lead to poor DSS in ACC, and improved DSS in KIRC (Figure 3J–3L).

The results of OS, DSS, and PFI indicated that upregulation of HOXD1, HOXD3, and HOXD4 was associated with poor ACC and improved KIRC. And the HOXD1, HOXD3, and HOXD4 played the role of oncogene or inhibitor in regulating of progression and metastasis of tumors.

### HOXD3 is down-expressed in KIRC tissues and cell lines

**Figure 4:**
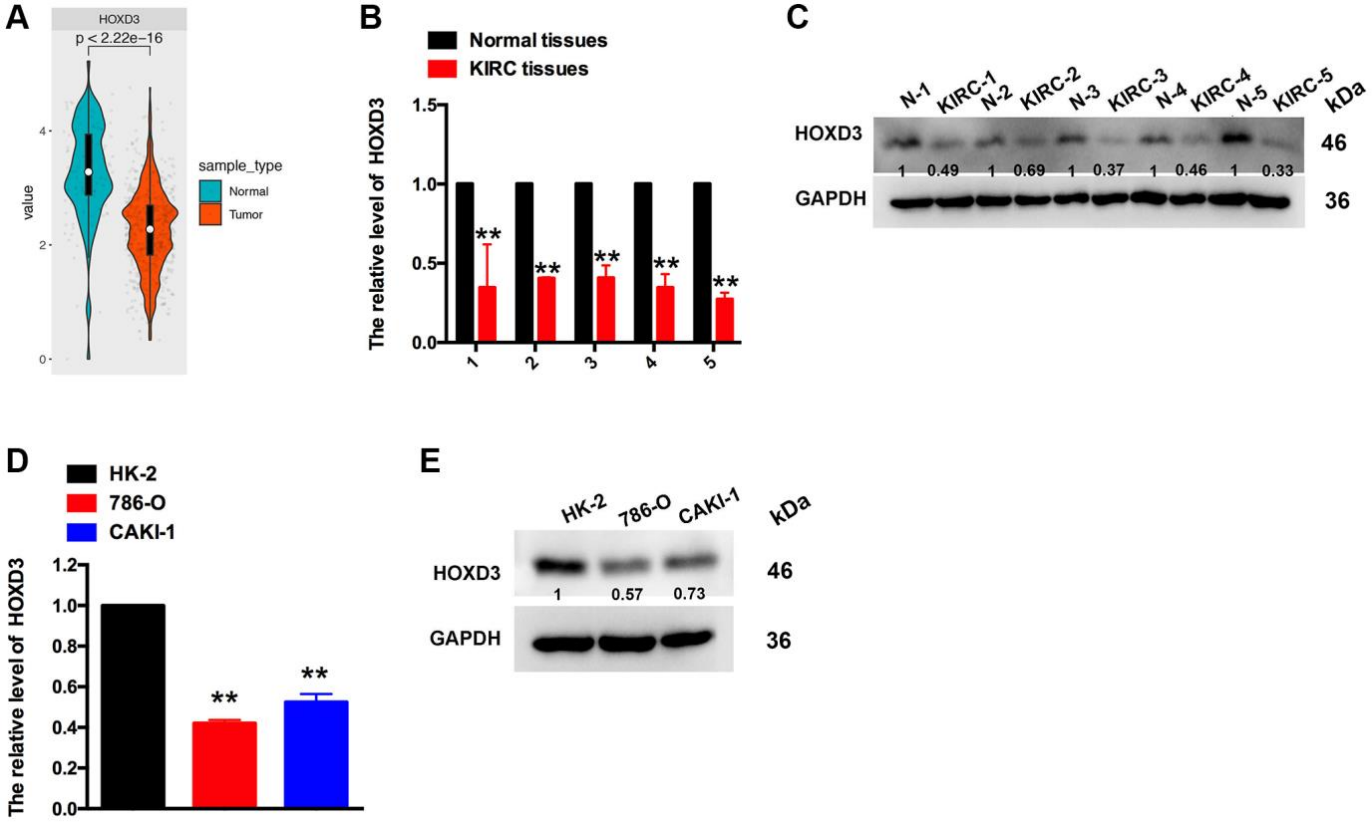
**The expression of HOXD3 in KIRC tissues and cell lines.** (**A**) HOXD3 expression was suppressed in KIRC tumor samples compared to normal tissue in the TCGA database. (**B**, **C**) The HOXD3 mRNA and protein levels were evaluated by qRT-PCR and western blotting in the KIRC tumor and normal tissue. (**D**, **E**) The HOXD3 mRNA and protein levels were identified by qRT-PCR and western blotting in 786-O, CAKI-1, and HK-2 (^**^p < 0.01).

The above results revealed that HOXD3 was down-expressed in KIRC tissues, compared with the normal tissues. The promotion of HOXD3 was connected with the improved OS, DSS, and PFI. Meanwhile, HOXD3 was negatively associated with the clinical stage and histological grade of KIRC. So, to identify the HOXD3 expression in KIRC tissues and cell lines further, the assays of qRT-PCR and western blotting were used. The outcomes revealed that HOXD3 was lower expressed in KIRC tissues, compared with non-cancerous tissues (Figure 4B, 4C), which was in line with the results in TCGA and GTEX databases (Figure 4A). Meantime, the expression of HOXD3 in CAKI-1 and 786-O cells was markedly lower than that of the HK-2 cells (Figure 4D, 4E), suggesting that HOXD3 could play the role of inhibitor in the progression of KIRC.

### Enhanced HOXD3 inhibits KIRC tumor proliferation, metastasis, and invasion

**Figure 5:**
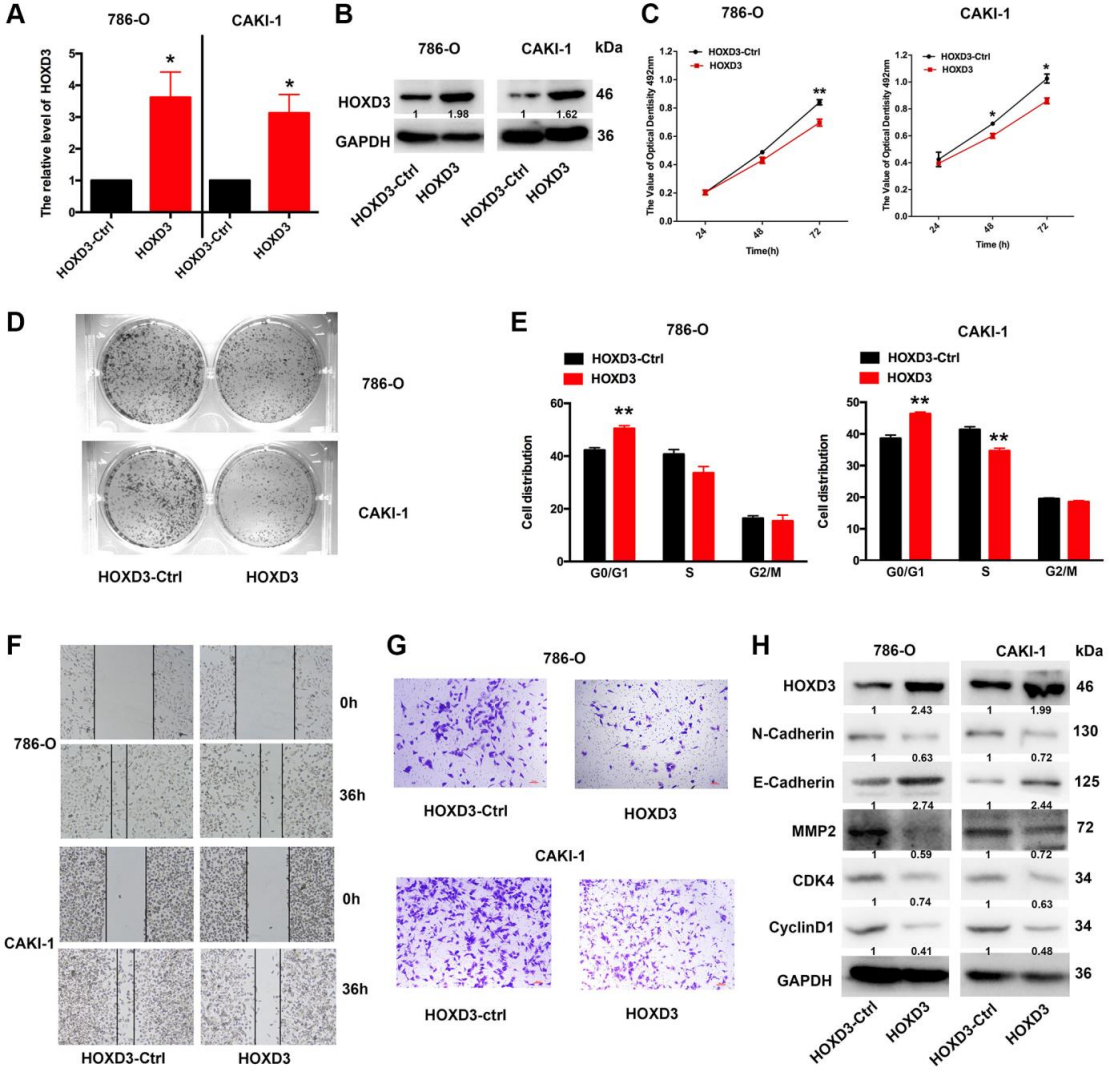
**HOXD3 overexpression inhibits proliferation in ccRCC cells.** (**A**, **B**) The expression level of HOXD3 at mRNA and protein levels was assessed by qRT-PCR and western blotting when 786-O and CAKI-1 cells were transfected with HOXD3-Ctrl and HOXD3. (**C**) The cell viability of ccRCC cells was examined by MTT assay at 24 h, 48 h, and 72 h. (**D**) Clone formation assay was used to test the colonies number of 786-O and CAKI-1 cells which were transfected with HOXD3-Ctrl and HOXD3. (**E**) Cell cycles were determined in 786-0 and CAKI-1 cells after transfection of the HOXD3 overexpression vector or HOXD3-Ctrl. The histogram indicates the percentage of cells at G0/G1, S, and G2/M cell-cycle phases. (**F**, **G**) The wound healing and transwell assays were applied for verifying the migration and invasion ability of HOXD3 and control-transfected human ccRCC. Scale bar, 100 μm. (**H**) The expression of N-cadherin, E-Cadherin, MMP2, CDK4, and CyclinD1 was demonstrated by western blotting in both 786-O and CAKI-1 cells of HOXD3 overexpression (*p* < 0.01).

To further investigate whether overexpression of HOXD3 in ccRCC cells affects tumor proliferation *in vitro*, HOXD3 plasmid was used to overexpress HOXD3 in ccRCC cells. At first, the overexpression efficiency was validated using qRT-PCR and western blotting (Figure 5A, 5B). Subsequently, the proliferation of ccRCC cells was identified via MTT, clone formation, and cell cycle assays. As a result, MTT showed that the proliferation of ccRCC cells was remarkably suppressed after HOXD3 overexpressing (Figure 5C). What’s more, the colony numbers were markedly decreased in HOXD3 transfected cells compared with control transfected cells (Figure 5D). Furthermore, cell cycle analysis illustrated that HOXD3 overexpression arrested G1-S transition, which further validated that HOXD3 suppressed the proliferation of ccRCC cells (Figure 5E). In addition, wound healing and transwell invasion assays illustrated that HOXD3 could suppress the migration and invasion of ccRCC cells (Figure 5F, 5G). The expression of N-Cadherin, MMP2, CDK4, and cyclinD1 was decreased at the protein level and E-Cadherin was increased (Figure 5H) when HOXD3 was transfected in ccRCC cells. Collectively, these findings showed that HOXD3 promoted the inhibitor role of ccRCC cells.

### Inhibition of HOXD3 increased KIRC tumor proliferation, metastasis, and invasion

**Figure 6:**
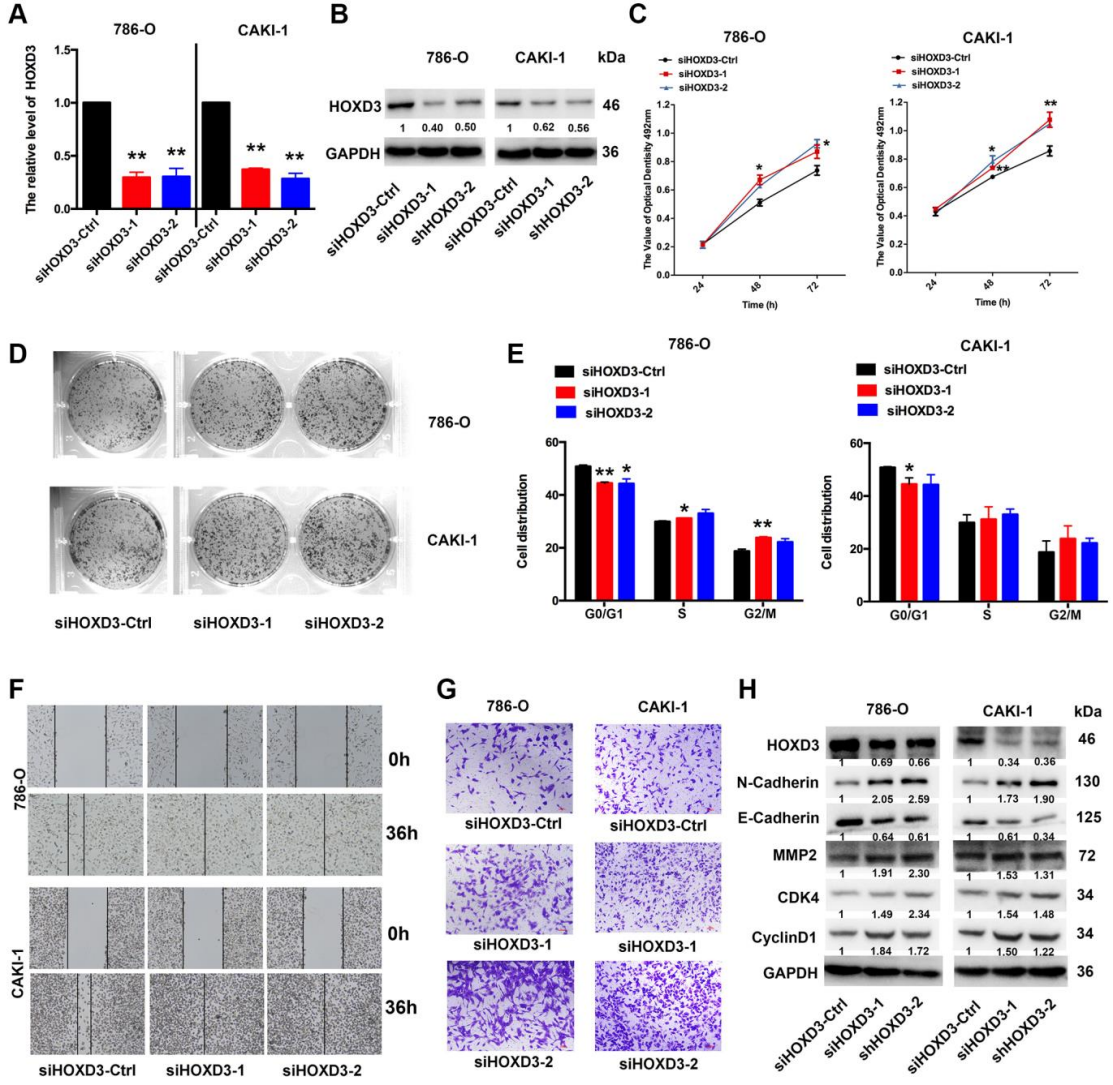
**HOXD3 down-expression induces proliferation in ccRCC cells.** (**A**, **B**) 786-O and CAKI-1 cells were transfected with siHOXD3-Ctrl, siHOXD3-1, and siHOXD3-2, and the HOXD3 expression at mRNA and protein levels was assessed by qRT-PCR and western blotting. (**C**) The effects of siHOXD3 on 786-0 and CAKI-1 cells viability were identified by MTT assay after transfecting of siHOXD3-Ctrl, siHOXD3-1 and siHOXD3-2. (**D**) Representative results of colony formation of 786-O and CAKI-1 cells after transfection of siHOXD3-Ctrl, siHOXD3-1, and siHOXD3-2. (**E**) Cell cycles were determined in 786-0 and CAKI-1 cells after transfection of siHOXD3-Ctrl, siHOXD3-1, and siHOXD3-2. (**F**, **G**) The wound healing and transwell assays were used to confirm the migration and invasion ability of siHOXD3 and control-transfected human ccRCC. Scale bar, 100 μm. (**H**) The expression of N-cadherin, E-Cadherin, MMP2, CDK4, and CyclinD1 was demonstrated by western blotting in both 786-O and CAKI-1 cells of HOXD3 down-expression (*p* < 0.01).

To investigate the role of HOXD3 in cell proliferation, metastasis, and invasion of ccRCC cells, silenced HOXD3 expression in 786-O and CAKI-1 cells using siRNA, and the HOXD3 expression was tested by qRT-PCR and western blotting (Figure 6A, 6B). The MTT assay revealed that downregulated HOXD3 increased the growth of ccRCC cells (Figure 6C). The colony numbers were markedly enhanced in siHOXD3 transfected cells compared with negative control cells (Figure 6D). Furthermore, cell cycle analysis illustrated that HOXD3 silencing induced the ccRCC cells at the G_0_ stage, (Figure 6E). What’s more, the suppression of HOXD3 enhanced the migratory and invasive capacities of 786-O and CAKI-1 cells (Figure 6F, 6G). Furthermore, the expression of N-Cadherin, MMP2, CDK4, and cyclinD1 was significantly elevated, and E-cadherin was inhibited in ccRCC cells that knocked down HOXD3 (Figure 6H), suggesting a tumor-suppressive role for HOXD3 in KIRC tumor.

### Enhanced HOXD3 inhibits KIRC tumor proliferation *in vivo*

**Figure 7:**
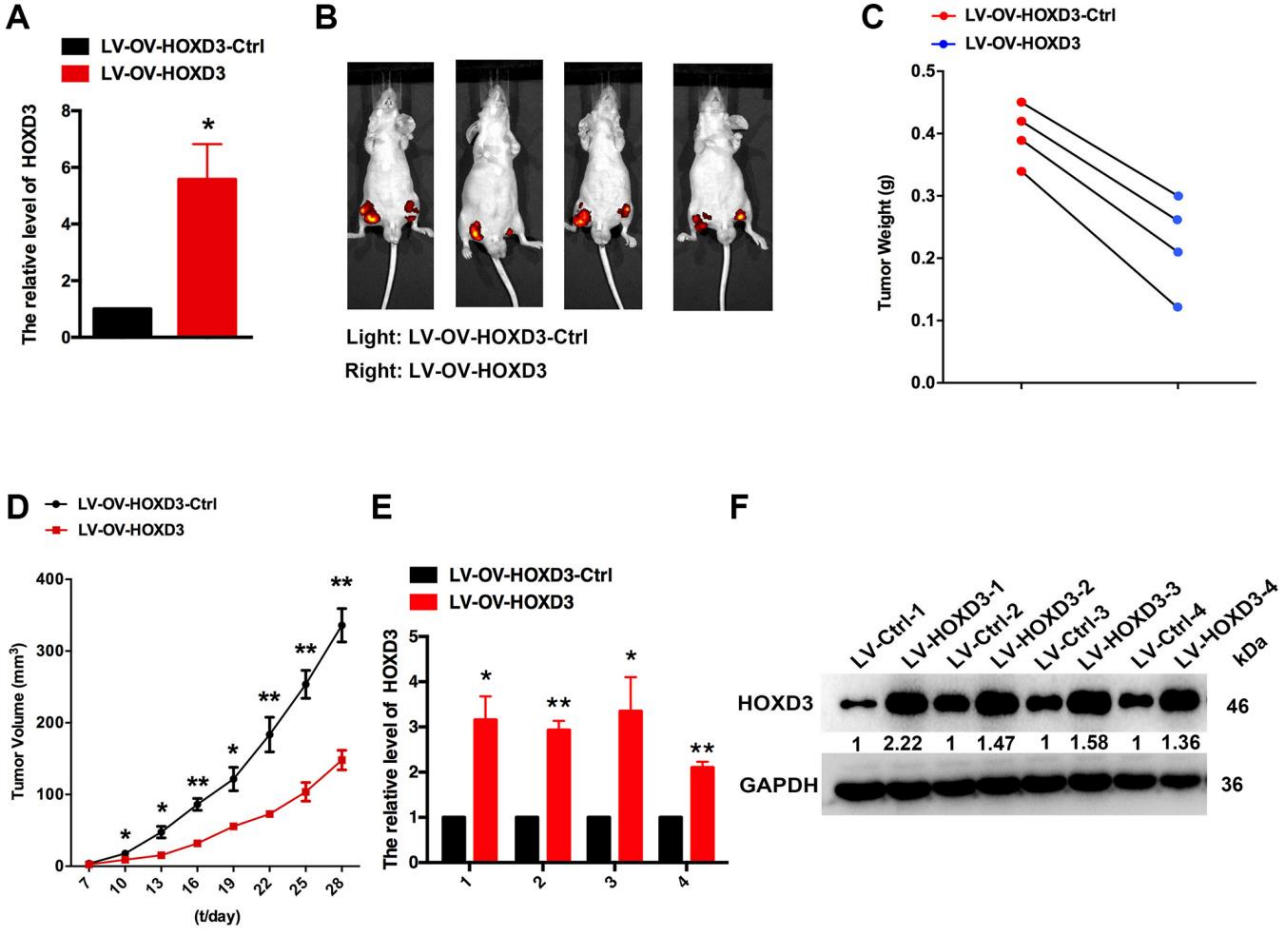
**HOXD3 inhibits KIRC progression *in vivo*.** (**A**) HOXD3 expression in LV-OV-HOXD3 and LV-OV-HOXD3-Ctrl cells was identified by qRT-PCR. (**B**) On day 28, the tumor was tested by bioluminescence imaging. (**C**) Tumor weight. (**D**) Tumor growth curves. (**E**, **F**) HOXD3 expression in tumor xenografts was confirmed by qRT-PCR and western blotting (^**^p < 0.01).

To demonstrate the function of HOXD3 in cell progression, the xenograft assay was performed. HOXD3 was highly expressed in CAKI-1 cells which were transfected with LV-OV-HOXD3 (Figure 7A). 1 × 10^6^ of LV- OV-HOXD3-Ctrl and LV-HOXD3- transfected CAKI-1 cells were inoculated to the nude mice. Tumors grown from HOXD3 stable overexpression cells were slower than tumors grown from the LV-HOXD3-Ctrl cells (Figure 7B). Overexpression of HOXD3 significantly reduced tumor volume tumor weight and size compared with LV-NC (Figure 7C, 7D). Expression of HOXD3 in tumor tissues was assessed by qRT-PCR and western blotting. The results demonstrated that the HOXD3 expression significantly was higher in LV-OV-HOXD3- infected tumors than in LV-Ctrl (Figure 7E, 7F). These results demonstrated that overexpression of HOXD3 efficiently suppressed the tumorigenesis of KRIC *in vivo*.

### Single-cell analysis of HOXD1, HOXD3, and HOXD4 in cancers

**Figure 8:**
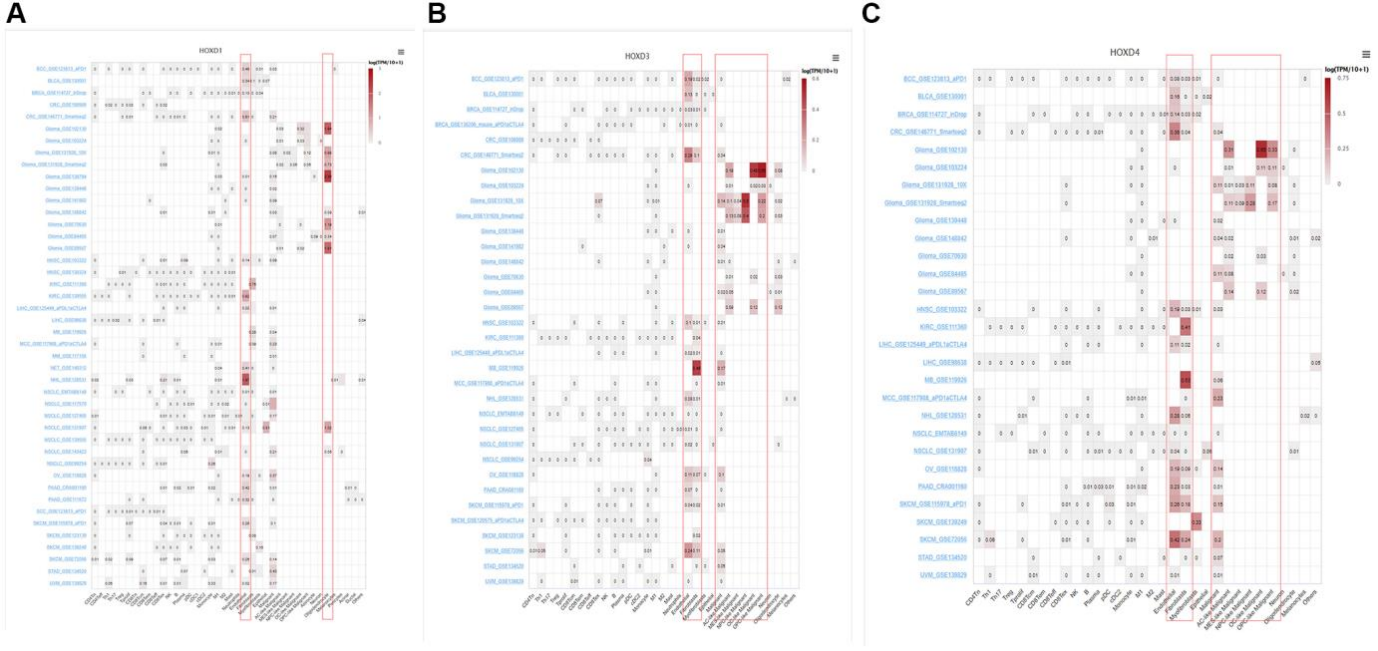
**Single-cell analysis of HOXD1, HOXD3, and HOXD4 in single-cell datasets of cancer samples.** (**A**) Summary of HOXD1 expression of 40 cell types in 44 single-cell datasets. (**B**) Summary of HOXD3 expression of 33 cell types in 34 single-cell datasets. (**C**) Summary of HOXD4 expression of 32 cell types in 29 single-cell datasets.

To identify the main cell types that express the HOXD1, HOXD3, and HOXD4 in cancer microenvironments, the single-cell analysis of HOXD1, HOXD3, and HOXD4 in single-cell datasets of cancer samples were used. The heatmap shown in Figure 8A and demonstrated the expression of HOXD1 of 40 cell types in 44 datasets using the TISCH. The results indicated that HOXD1 was mainly expressed in endothelial and oligodendrocyte cells; HOXD3 in endothelial, fibroblasts, and malignant cells (Figure 8B); HOXD4 in endothelial, fibroblasts, and malignant cells (Figure 8C). There is the high expression in Glioma_GSE138794 of HOXD1, five cell types were found, including astrocyte, endothelial, malignant, Mono/Macro, and oligodendrocyte cells. And in Glioma_GSE102130 of HOXD3 and 4, five cell types were found, including AC-like malignant, Mono/Macro, OC-like malignant, OPC-like malignant, and oligodendrocyte cells (Supplementary Figure 1).

### GSEA of HOXD1, HOXD3, and HOXD4 in pan-cancer

**Figure 9:**
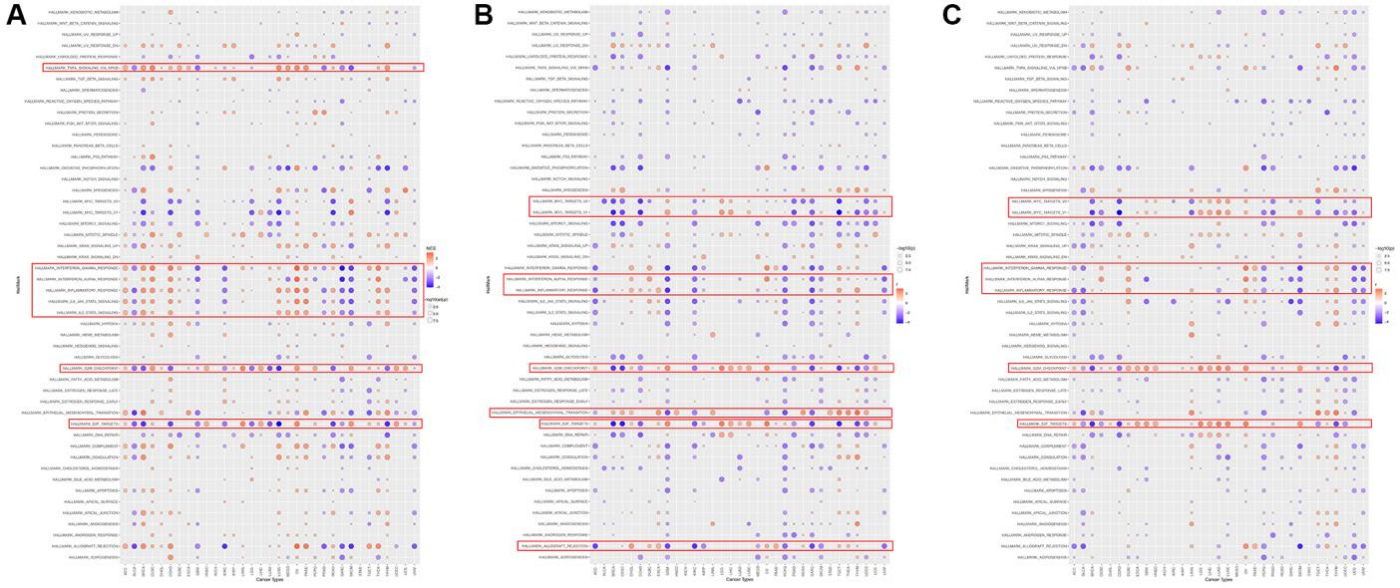
The hallmarks gene set enrichment analysis of HOXD1 (**A**), HOXD3 (**B**), and HOXD4 (**C**) in pan-cancer.

GSEA was performed to assess the signaling pathways of HOXD1, HOXD3, and HOXD4 that cause their differential expression in each cancer. The result showed that expression of HOXD1 was significantly associated with pathways, such as TNFA-signaling-via-NFKB, IFN-γ response, IFN-α response, inflammatory response, IL6-JAK-STAT3, IL2-STAT5, G2M-checkpoint and E2F-targets pathways (Figure 9A). HOXD3-related pathways were Myc-targets-V1/2, IFN-α response, inflammatory-response, G2M-checkpoint, mesenchymal-transition, E2F-targets and allograft-rejection pathways (Figure 9B). HOXD4-related pathways were Myc-targets-V1/2, IFN-γ response, IFN-α response, inflammatory-response, G2M-checkpoint, and E2F-targets pathways (Figure 9C). Above all, these results indicated that HOXD1, HOXD3, and HOXD4 expression was significantly enriched with pathways associated with the immune-activation status and progression of cancers.

### TIMER immune cell infiltration analyses

**Figure 10:**
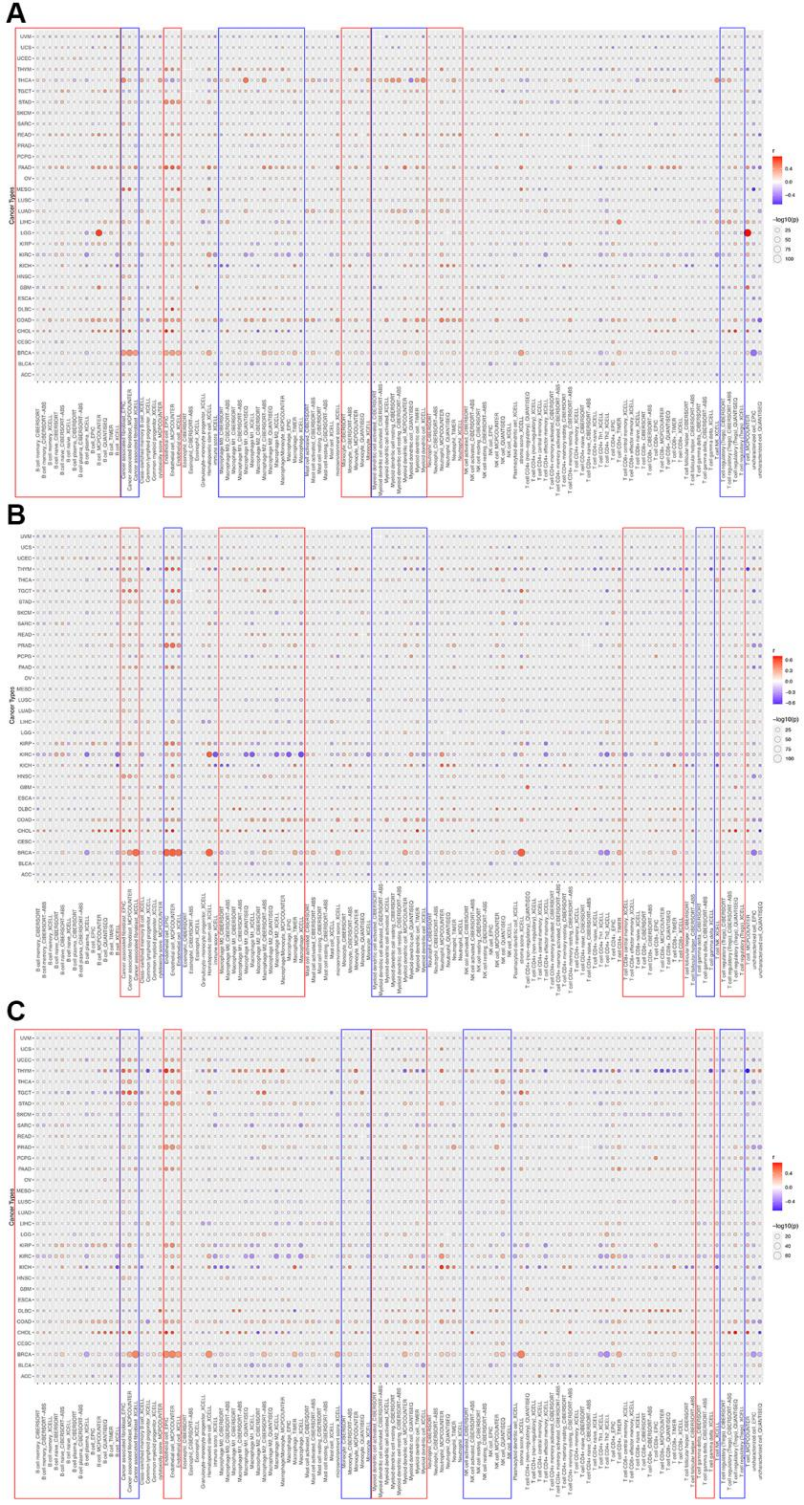
The correlations of HOXD1 (**A**), HOXD3 (**B**), and HOXD4 (**C**) expression and the infiltration levels of CD4+ T cells, CAF, progenitor, Endo, Eos, HSC, Tfh, γδT, regulatory T cells (Tregs), B cells, neutrophils, monocytes, macrophages, dendritic cells, NK cells, Mast cells and CD8+ T cells in cancers.

To determine the correlation between HOXD1, HOXD3, and HOXD4 and cancer immunity, the TIMER database was used. The outcomes showed that HOXD1 was positively connected with the infiltration levels of B cells, CAF, Endo, macrophages, monocytes, neutrophils, dendritic cells, and Tregs in most of the cancers (Figure 10A). Whereas, HOXD1 was negatively associated with the infiltration level of most immune cells such as the B cells, CAF, Endo, macrophages, Mast, neutrophils, monocyte, NK, CD8+ T, Tfh, and dendritic cells in UVM. In addition, HOXD3 was positively associated with the CAF, Endo, macrophages, dendritic, CD8+ T, and Tregs cells in most of the cancers and negatively correlated with the γ/δT cells in most cancers, especially in KIRC (Figure 10B). HOXD4 was positively associated with the B cells, CAF, Endo, monocytes, dendritic, NK, and Tregs cells in most of the cancers and negatively associated with the γ/δT cells in most cancers except UVM, THCA, GBM, ESCA, DLBC cancers (Figure 10C).

## DISCUSSION

HOXDs belong to the HOX family, and it plays a crucial role in enhancing/suppressing cancer cells by modulating cancer-related oncogenes or inhibitor. HOXD1, HOXD3, and HOXD4 were located at the 3′ end of the HOXD cluster and decided the development of head and neck vertebrae [17, 18]. HOXD1, HOXD3, and HOXD4 were transcription factors and have been recognized as oncogenes in many cancers, such as liver cancer, gastric cancer, breast cancer, and as an inhibitor in kidney renal clear cell carcinoma [15]. Whereas, the molecular function of HOXD1, HOXD3, and HOXD4 in tumorigenesis, recurrence, and metastasis of various cancers have still unknown. What’s more, there has been no research referring to their effect on pan-cancer.

In varying degrees, HOXD1, HOXD3, and HOXD4 participated in the initiation and progression of the tumor. Based on these results, the correlation between the expression of HOXD1, HOXD3, and HOXD4 and the cancer pathogenesis, underlying mechanisms, and tumor immunity were studied in this research, which was the first comprehensive analysis of the differential expression and related mechanisms of HOXD1, HOXD3, HOXD4 in various cancers.

Here, the expression of the HOXD1, HOXD3, and HOXD4 genes in 33 tumors in the TCGA database was analyzed. Results confirmed that differential expression of HOXD1, HOXD3, and HOXD4 between cancer tissues and normal tissues existed in many types of cancer. Based on the data showed HOXD1 was increased in ACC, CESC, DLBC, ESCA, HNSC, CHOL, LGG, LIHC, LUAD, OV, PAAD, SKCM, STAD, THCA, THYM, UCEC, and UCS (*p* < 0.01). However, the opposite results had been demonstrated in HBV-HCC and breast cancer. In the HBV-HCC, highly expressed IGF2-AS could suppress the development of HBV-HCC by upregulating HOXD1 [19]. In addition, 3′UTRs of HOXD inhibited migration, invasion, and adhesion of breast cancer via serving as STARD13 ceRNAs [20]. Meantime, the analysis of HOXD1 expression in TCGA databases revealed the downregulation of this gene in tumor tissues from BRCA, COAD, KICH, KIRC, KIRP, READ, and TGCT (Figure 1). The above result was confirmed in the KIRC study, the study showed that HOXD1 is lowly expressed in KIRC and as a suppressor to inhibit the TGF-β signaling to regulate the cell proliferation [15].

Furthermore, the expression of the HOXD3 gene in 33 tumors in the TCGA was analyzed. Compared with normal tissues, HOXD3 had a lower expression level in BRCA, CESC, COAD, KICH, KIRC, KIRP, PRAD, READ, TGCT, THCA, UCEC, UCS (*p* < 0.01). Meantime, high expression was observed in CHOL, DLBC, GBM, HNSC, LGG, LIHC, LUAD, LUSC, PAAD, PCPG, SKCM, STAD, and THYM (*p* < 0.01). Consistent with our previous, using the RT-PCR and western blotting assays, the results showed that HOXD3 was highly expressed in LIHC [16]and also confirmed in the gastric cancer [21]. Mechanistically, vitamin D receptor (VDR) – mediated miR-99b-3p targeted the HOXD3 to inhibit the proliferation of gastric cancer cells. Compared with normal breast tissue, HOXD3 was high-expressed in breast cancer tissue. The overexpression of HOXD3 increased breast cancer cell drug resistance through integrin β3-mediated Wnt/β-catenin signaling [22]. In contrast, our results demonstrated that HOXD3 was low expressed in KIRC, and overexpression of HOXD3 could decrease the ccRCC cells proliferation *in vivo* and *in vitro* (Figures 4–7), which was confirmed by using the TCGA database. Meantime, Wang, et al. showed that the HOXD3 was hyper-methylated in ccRCC samples [23], which could explain the reason, to some extent, why the HOXD3 was low-expressed in KIRC. Meantime, HOXD3 represented high methylation levels found in prostate cancer patients [24], which was associated with the recurrence of prostate cancer [25]. This suggests that HOXD3 plays a different role in various cancers.

Next, the expression of HOXD4 in various cancers was evaluated in the TCGA database. HOXD4 was overexpressed in CHOL, DLBC, GBM, HNSC, LGG, LIHC, LUAD, LUSC, PAAD, SKCM, and THYM (*p* < 0.01). Inhibition of HOXD4 expression was shown in ACC, BRCA, CESC, COAD, ESCA, KICH, KIRC, KIRP, OV, PRAD, READ, STAD, TGCT, THCA, UCEC, and UCS (*p* < 0.01) (Figure 1). Most of the results were consistent with former studies [8, 26, 27]. In gastric adenocarcinoma, highly expressed HOXD4 is associated with positive lymph node metastasis and enhanced proliferation of gastric adenocarcinoma by increasing c-Myc and cyclin D1.

Pathological stage, histological grade, and immune subtype were used to reveal the prognostic value of the tumor. According to the analysis in KIRC and LIHC, HOXD1, HOXD3, and HOXD4 expressions were associated with histological grade. In the KIRC, the expression of HOXD3 decreased as the histological grade escalated. However, in the LIHC, the histological grade was positively correlated with HOXD3 expression (Figure 2). In our previous, we found that expression of HOXD3 was extremely connected with tumor histology and tumor stage in LIHC [16].

Furthermore, in the KIRC, all HOXD1, HOXD3, and HOXD4 expressions were negatively linked with the tumor stage. We further performed the experimental verification of HOXD3 in KIRC to prove its suppressor role. The results showed that overexpression of HOXD3 could suppress the proliferation, invasion, and migration of 786-O and CAKI-1 cells (Figure 5). Whereas, inhibition of HOXD3 increased these effects of HOXD3 in 786-O and CAKI-1 cells (Figure 6). These results demonstrated that HOXD3 was a key gene that inhibits the progression of KIRC.

Kaplan-Meier survival analysis demonstrated that high HOXD3 expression was linked to poor prognosis in LIHC. Similarly, expression of HOXD3 was previously demonstrated as associated with shorter survival time in patients with liver cancer [16]. Moreover, Cheng reported that HOXD3 expression is a significant unfavorable prognostic factor and could serve as a potentially useful prognostic indicator for patients with invasive breast cancer [28]. In contrast, low HOXD3 expression is associated with good prognosis in patients with KIRC, contained with the outcomes of OS, DSS, and PFI (Figure 3), this result was confirmed in the upregulation of HOXD1 and HOXD4.

The GSEA result suggested that HOXD1, HOXD3, and HOXD4 are closely associated with the immune-related pathways, such as TNFA-signaling-via-NFKB, IFN-γ response, IFN-α response, inflammatory-response, IL6-JAK-STAT3, IL2-STAT5. Meantime, the GSEA result showed that HOXD1, HOXD3, and HOXD4 were connected with the immune infiltration in pan-cancer. Consistent with the above results, the cyclin D1b could be connected with HOXD3 inducing EMT to affect inflammatory microenvironment [29]. Furthermore, HOXD1, HOXD3, and HOXD4 are related to the processes of G2M-checkpoint and E2F-targets. In the research KIRC, HOXD1 was implicated in the regulation of cell cycle [15]. In addition, the study from Zha et al. revealed that HOXD3 could suppress cell proliferation through the S, G2, and M phases [30]. These results indicated that expression of HOXD1, HOXD3, and HOXD4 was associated with the immune microenvironment, providing a potential drug target for tumor immunotherapy.

## CONCLUSIONS

Our first pan-cancer analyses of HOXD1, HOXD3, and HOXD4 indicate that those factors were differentially expressed between normal and tumor tissues and reveal associations of gene expression with clinical indicators. Our findings suggest that HOXD1, HOXD3, and HOXD4 expression will bring different prognostic outcomes, which needs to be further studied for the specific role of HOXD1, HOXD3, and HOXD4 in each cancer. Moreover, HOXD1, HOXD3, and HOXD4 could be applied to evaluate immune cells’ infiltration in various tumor tissues. These findings may help to unveil the effect of HOXD1, HOXD3, and HOXD4 on tumorigenesis and development, and can provide a reference for further investigation of the HOXD family genes as potential therapeutic cancer targets in pan-cancer.

## Supplementary Materials

Supplementary Figure 1
