# Supplementary figures and images for: A pan-cancer analysis of the role of HOXD1, HOXD3, and HOXD4 and validation in renal cell carcinoma
Source: Aging (Albany NY). 2023 Oct 12;15(19):10746–66. doi: 10.18632/aging.205116 (PMC10599751; doi:10.18632/aging.205116)

SUPPLEMENTARY FIGURE

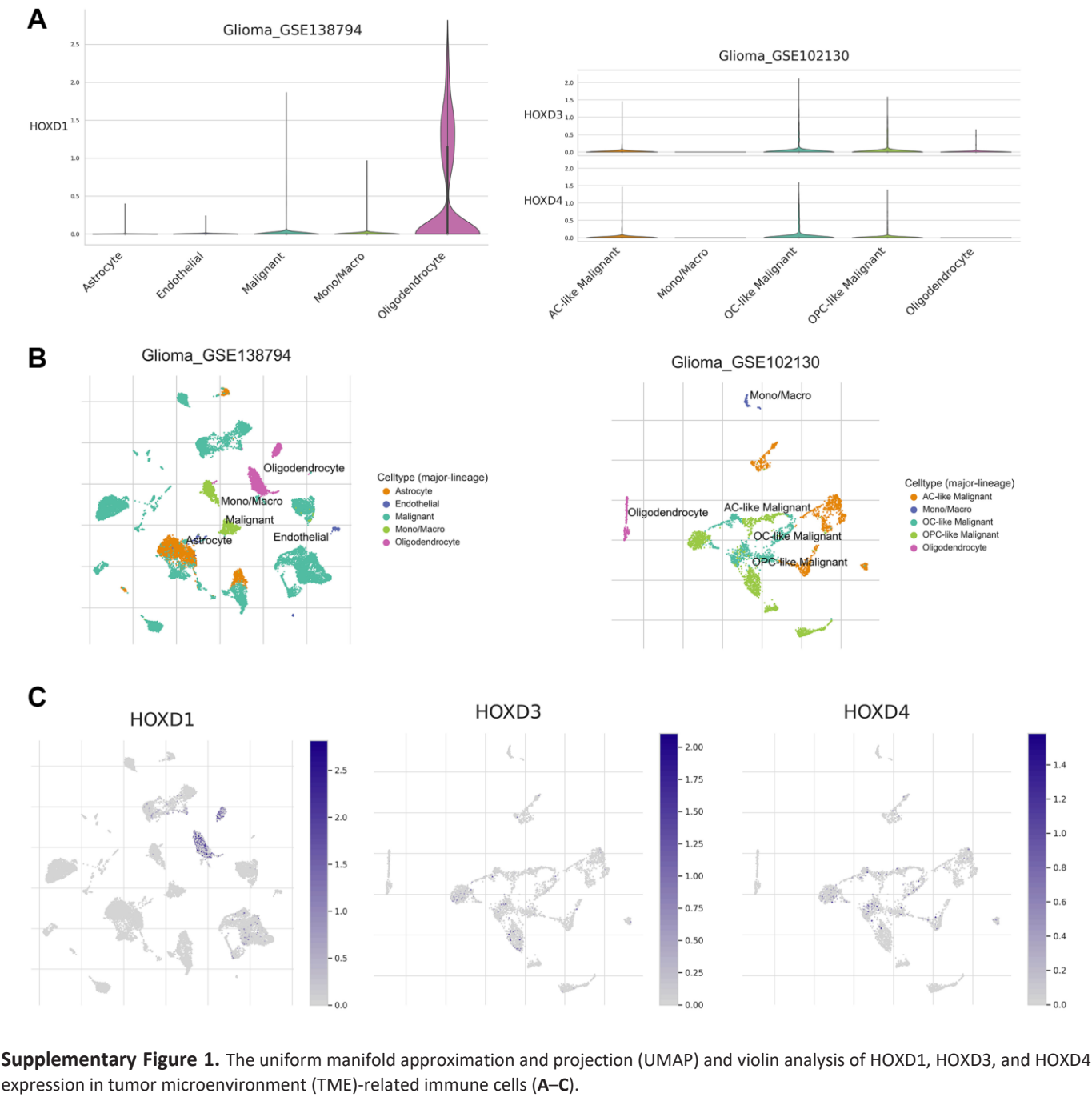

Supplement: Supplementary Figure 1 [file aging-15-205116-s001.pdf]
